# General paediatrics: motives and concerns of medical students on the career pathway: Lenka Šlegerová

**DOI:** 10.1093/eurpub/ckac130.149

**Published:** 2022-10-25

**Authors:** L Šlegerová, P Michenka, M Kočí

**Affiliations:** 1 Institute of Economic Studies, Charles University, Faculty of Social Sciences, Prague, Czechia; 2 Charles University, Third Faculty of Medicine, Prague, Czechia; 3 Charles University, Second Faculty of Medicine, Prague, Czechia

## Abstract

Child public health is dependent on preventive care provided by primary care physicians for children and adolescents. Nevertheless, their accessibility in Czechia is exceedingly endangered by their ageing (the average increased by 1.5 years to 57.5 in eight years). This study aims to (1) identify medical students who opt for paediatric residency and explore the effect of undergraduate training on their decision, (2) detect factors that would persuade them to become practitioners, and (3) factors influencing their choice of a medical facility. The analysis builds on our survey's unique data exploring students’ views on training and occupational preferences. Students in the fourth to the sixth year of all medical faculties in Czechia were addressed in two waves (2020 and 2021) of this cross-sectional study, resulting in 2,283 complete questionnaires (response and coverage rate of 25.3%). Out of these, 306 respondents have already decided on or shortlisted paediatrics as a residency speciality, with a strong over-representation of women (85.3%). The evaluation of undergraduate paediatric training's quality (in terms of its organisation, number of students and teachers’ feedback) is mildly correlated with considering the paediatric residency (Cramer's V < 0.1). Fewer administrative obligations represent the most frequent factor necessary to lean towards primary care, reported by 54% of respondents preferring hospital paediatrics (or yet undecided); a financial entry bonus is less convincing (only 27% of them). To attract graduates, medical facilities need to provide favourable conditions for residency (good supervisor, employer's support in the training); financial offers might not be an adequate decoy in less-attractive areas. This survey sheds light on medical students’ views and decision-making processes, which are essential to consider in the public discussion on the future accessibility of primary care for children and adolescents.

**Key messages:**

- To captivate graduates, medical facilities need to provide favourable conditions for residency (good supervisor, employer's support in the training); financial offers might not be sufficient.
- Fewer administrative obligations laid upon primary care physicians are necessary to attract medical students to general paediatrics to reverse the ageing trend of practitioners in Czechia.

